# Implementation Outcomes and Their Determinants for Hospital‐Led Care Coordination Interventions Targeting Patients With Complex Care Needs: A Qualitative Systematic Review

**DOI:** 10.1111/jocn.70102

**Published:** 2025-09-15

**Authors:** Mary Malakellis, Anna Wong Shee, Sarah Wood, Laura Alston, Hannah Beks, Margaret Murray, Vincent L. Versace, Kevin Mc Namara

**Affiliations:** ^1^ Deakin Rural Health Deakin University Warrnambool Victoria Australia; ^2^ Grampians Health Ballarat Victoria Australia; ^3^ Colac Area Health Research Unit Colac Victoria Australia

## Abstract

**Aim:**

To describe the implementation determinants for care coordination interventions in a hospital context.

**Design:**

Systematic review.

**Methods:**

This review was guided by the Consolidated Framework of Implementation Research (CFIR), assessed for quality using the Mixed Methods Appraisal Tool and reported with the PRISMA guidelines.

**Data Sources:**

CINHAL Complete, EMBASE, MEDLINE Complete, PsychINFO (between January 1, 2013, and December 31, 2022, and updated May 09, 2024) and a manual reference list search of all included studies.

**Results:**

The search returned 5614 articles after duplicates were removed. After title and abstract screening, 264 articles underwent full‐text review. Sixteen studies (15 care coordination models) met the inclusion criteria. The CFIR inner setting domain and the implementation process domain were the most prominent domains and ‘Partnerships & Connections’, ‘Work Infrastructure’, ‘Capability’ and ‘Reflecting and Evaluating’ subdomains emerged as important determinants across the included studies.

**Conclusion:**

Inconsistent findings relating to care coordination outcomes are likely to be substantially influenced by the complexity and heterogeneity of the interventions and variations in implementation and contextual factors. Intra‐ and inter‐organisational relationships were important to connect previously disconnected parts of the health system and were facilitated by experienced care coordinators. Continual improvement was also important to increase fit with contextual factors. More high‐quality studies are needed to identify commonalities and provide generalisable principles and characteristics associated with high‐performance implementation.

**Implications for the Profession and/or Patient Care:**

Review findings will provide practitioners, policymakers, and researchers with a comprehensive synthesis of evidence underpinning implementation of effective community care coordination from hospital settings.

**Impact:**

These review findings will inform the effective implementation of care coordination interventions in a hospital context for patients with complex multimorbidity.

**Reporting Methods:**

Preferred Reporting Items for Systematic reviews and Meta‐Analysis.

**Trial and Registration:**

PROSPERO Registration: CRD42022376642.

**Patient or Public Contribution:**

No patient or public Contribution.

**Summary**

- What does this paper add to the wider global clinical community?
  ○ The review identifies evidence of effective implementation strategies for care coordination interventions to address people with complex and chronic conditions.
  ○ Contextual features have been identified which enable or constrain implementation of care coordination interventions in a hospital context and may influence scale‐up in similar settings.
  ○ Clear descriptions of care coordination interventions, the care coordinator role, and the macro‐context to facilitate generalisability and transferability are needed in future studies.

## 1 Introduction

In developed countries, patients with complex chronic conditions account for a disproportionate share of national health spending (Hofmarcher et al. 2007; Schoen et al. 2011). Care delivery for these patients often involves multiple health professionals and healthcare services, and coordinating this care has proven challenging (Mc Namara et al. 2017). Failures of care coordination are particularly common as patients move between primary care and specialty care, at hospital discharge, and in settings such as outpatient clinics and the emergency department (ED) (Hofmarcher et al. 2007; Schoen et al. 2011). Poor care coordination has been associated with adverse patient outcomes including increased healthcare use, healthcare costs (Joo 2023) and poor patient experiences (Gadbois et al. 2019). Care coordination is difficult to achieve because typically healthcare delivery or programs have evolved in ‘silos’ or settings that are independent, with different organisational structures, processes and priorities (Hofmarcher et al. 2007). Improving care coordination is critical for providing timely, effective, safe, efficient and equitable patient‐centred care (Kizer 2015). Increases in health system complexity and prevalence of complex chronic conditions have led to an increase in dedicated roles, such as care coordinators, to address barriers and improve care. Care coordination interventions are widely recognised as important to achieve patient‐centred healthcare and to reduce potentially unnecessary use of acute care services (e.g., ED visits, hospital admissions) for patients with complex chronic conditions (Hofmarcher et al. 2007; Bodenheimer 2008; Corrigan and Adams 2003).

Care coordination is a comprehensive approach to facilitate the appropriate delivery of healthcare, improve continuity and bridge transitions of care (e.g., transfer of care between providers) (Schultz and McDonald 2014). The broad concept of care coordination incorporates a variety of strategies including case or care management, care integration and patient or nurse navigation (Schultz and McDonald 2014). Several systematic reviews have reported inconsistent effects of care coordination interventions on reducing hospital admissions, ED presentations, length of stay and healthcare costs (Buja et al. 2020; Duan‐Porter et al. 2022; Joo and Huber 2019). The lack of consistent results may relate to the complexity and heterogeneity of the interventions and variations in implementation and contextual factors (Buja et al. 2020; Duan‐Porter et al. 2022; Joo and Huber 2019). Care coordination is a complex and multifaceted intervention characterised by interacting components and organisational systems, variations in delivery, a range of goals, and that require flexibility and tailoring for successful implementation (Craig et al. 2011). As such, effects of care coordination interventions depend on contextual factors and how well they are implemented (Albertson et al. 2022; Hudon et al. 2017; Kokorelias et al. 2021).

While care coordination interventions are increasingly being used nationally and internationally, there is a lack of evidence around factors that enable their effective and sustainable implementation in a hospital context—particularly for patients with complex multimorbidity where care coordination relies on connecting with multiple community‐based health and social services. Effective implementation is associated with better outcomes (Durlak and DuPre 2008), and previous research has demonstrated that contextual factors have a substantial impact on implementation (Moore et al. 2021). Further, a better understanding of how to implement care coordination interventions in hospital settings may enhance transferability. The primary aim of this review was to describe the implementation determinants for care coordination interventions in a hospital context.

## 2 Methods

A systematic review was undertaken to describe, critically appraise and synthesise the literature according to the Consolidated Framework for Implementation Research (CFIR) (Damschroder et al. 2022). See The PRISMA 2020 statement: An updated guideline for reporting systematic reviews (Appendix S1). This review has been registered with the International Prospective Register of Systematic Reviews (PROSPERO CRD42022376642) and complies with the Preferred Reporting Items for Systematic Reviews and Meta‐Analyses statement (Moher et al. 2009). A review protocol was not prepared for this review.

### 2.1 Search Strategy

The search strategy was developed by identifying concepts from the research question, identifying synonyms for each concept, adding search techniques to each term and using gold set articles to test the search. A librarian with specialist training in performing systematic reviews peer reviewed the search strategy. Studies were identified using the following electronic databases: CINAHL Complete, Embase, MEDLINE Complete, and PsychINFO. The search was conducted on January 19, 2023 and rerun on May 09, 2024. The search was limited to studies published in the English language between January 1, 2013 and December 31, 2022, and extended to May 09, 2024, to reflect recent implementation science frameworks, current hospital settings and the latest policies. The search strategy included selected Medical Subject Headings and key words adapted to each database and represented three main concepts: care coordination models, hospital settings and implementation (for the full search strategy, see Appendix S2). The reference list of studies that met the inclusion criteria was assessed for additional eligible articles for inclusion in the review.

### 2.2 Operational Definition of Care Coordination

Ambiguity remains about the definition of care coordination with a review identifying 57 unique definitions of care coordination (Schultz and McDonald 2014). Further, the most common care coordination intervention, case management, is often poorly described with mixed concepts and constructs and with no consensus on what is and what is not case management (Lukersmith et al. 2016). Similarly, there is significant ambiguity in the role and function of a case manager, the term used interchangeably in the literature with for example, patient navigators, with both described to have overlapping functions (Kelly et al. 2019). Given the heterogeneity, complexity and inadequate descriptions of care coordination interventions, a flexible exploratory approach with consideration of the breadth of literature encompassing a range of interventions was adopted. We sought to include holistic interventions that embraced the case management definition ‘a collaborative process of assessment, planning, facilitation and advocacy for options and services to meet an individual's holistic needs through communication and available resources to promote quality cost‐effective outcomes’ (Marfleet and Trueman 2013).

### 2.3 Selection Criteria

The inclusion criteria for the studies were: (1) the interventions described were a care coordination model (e.g., case management, nurse/patient navigation) supporting referrals for patients (aged ≥ 18 years) to meet social and healthcare needs; (2) the intervention coordinator was based in a hospital setting; and (3) the focus of the research was related to implementation strategies (Powell et al. 2015), implementation determinants (Damschroder et al. 2022, 2009) or implementation outcomes (e.g., acceptability, adoption, appropriateness, cost, feasibility, fidelity, penetration, sustainability) (Proctor et al. 2011). The exclusion criteria for the studies were (1) the intervention was not described; (2) referrals were internal to an organisation (e.g., inpatient‐focused care coordination); (3) a focus on care of one condition (e.g., cancer only) or aspect of care (e.g., adherence to medication); and (4) care coordination with limited monitoring and follow‐up (e.g., single phone call or referral according to protocol).

### 2.4 Study Selection

Search results were imported to Covidence systematic review software (Veritas Health Innovation, Melbourne, Australia) to support the screening and assessment processes and duplicates were removed. Titles and abstracts of all identified studies were independently assessed by researchers (MMa, MM, SW, HB, LA, AWS, KM) for eligibility. Three researchers (MMa, MM, SW, HB) assessed the full texts of included studies if the title or abstract met the eligibility criteria. Disagreements between reviewers were resolved through discussion until consensus was reached.

### 2.5 Data Extraction

Microsoft Excel (Version: 16.90.2, Licence: Microsoft 365 subscription, Year: 2024) software was used for data extraction. Data describing study design, country, setting, number of sites, participant and intervention characteristics, implementation characteristics, implementation strategies and implementation outcomes of interest were extracted. Elements of care coordination interventions were extracted according to the Case Management Society of Australia and New Zealand Standards of Practice for Case Management and were operationalised for this review (Appendix S3). Given the heterogeneity of care coordination interventions and the role and function of a care coordinator (e.g., case manager, patient/nurse navigator), this framework was used to guide comparisons between studies. We referred to related articles (e.g., protocol) of studies where additional details of care coordination interventions were available. We interpreted real‐world studies as those conveying the health service as leading or funding the implementation of care coordination interventions as routine care and which were not highly controlled (e.g., all target patients were included) (Kim et al. 2018). Implementation determinants were extracted from the results section of included studies and categorised according to CFIR (Damschroder et al. 2022, 2009). The CFIR framework consists of 5 major domains: (1) innovation domain, (2) outer setting domain, (3) inner setting domain, (4) individuals domain and (5) implementation process domain. Each of these 5 domains is further divided into sub‐domains or constructs associated with implementation (further information available at http://www.cfirguide.org/constructs) (Damschroder et al. 2022, 2009). CFIR was chosen as the organising framework because it is commonly used and aids the transferability and comparability of findings from this review to other implementation studies (Damschroder et al. 2022, 2009). Two researchers (MMa and KM) independently piloted a structured data extraction tool on the same five articles before extracting individually for the remaining articles. Disagreements between reviewers were resolved through discussion until consensus was reached. A heat map was created depicting the count of extracted data for each domain and subdomain of CFIR.

### 2.6 Quality Assessment

As appropriate for individual study design, sections of the Mixed Methods Appraisal Tool (MMAT) version 2018 (Hong et al. 2018) were used to assess the quality of each article. Two reviewers (MMa, MM) independently appraised each study, with any discrepancies over the quality or risk of bias discussed and resolved.

### 2.7 Data Analysis

A synthesis without meta‐analysis was undertaken for this systematic review because of the heterogeneity of both the care coordination models implemented and implementation determinants, strategies and outcomes. Extracted data were coded directly against the CFIR constructs using framework synthesis (Brunton et al. 2020). Framework synthesis consists of five stages to help organise and bring complex components together in the review. The framework synthesis consists of five steps as follows: (1) familiarisation: becoming immersed in the data, (2) framework selection: identifying and selecting a framework, (3) indexing: coding extracted data according to the framework, (4) charting: summarising the data into categories and deriving themes directly from the data and (5) mapping and interpretation: interpreting and synthesising the data identified in reference to the original research question.

## 3 Results

**FIGURE 1:**
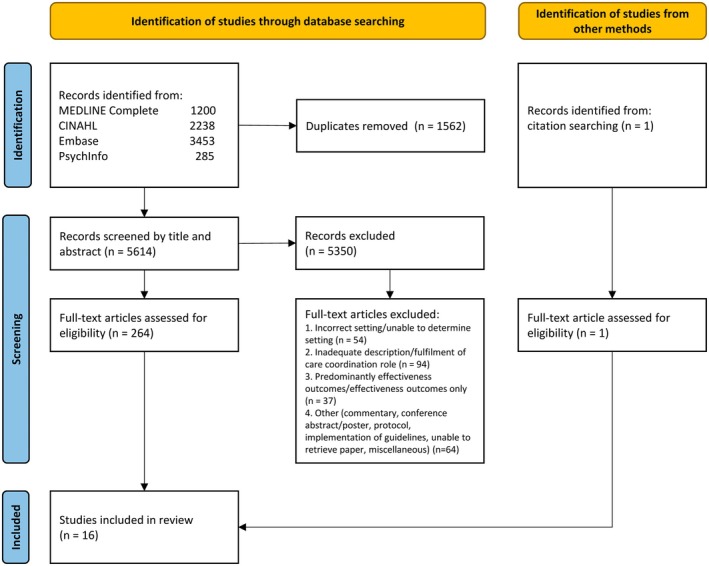
PRISMA flow diagram of study selection process. [Colour figure can be viewed at wileyonlinelibrary.com]

The search of the electronic databases retrieved 7176 records, of which 1562 were duplicates (Figure 1). Of the remaining 5614 records, 264 articles were retained after title and abstract screening, of which 15 met the inclusion criteria following full text eligibility assessment. An additional study was identified by screening the reference list of included articles, resulting in 16 eligible studies describing 15 care coordination models.

### 3.1 Quality Appraisal Results

The quality of reporting was variable between studies. All included articles had clear research questions and collected appropriate data to address the research questions. Areas of poorer quality included lack of rationale for the methodological approach, lack of reference to a theoretical framework, and reflexivity. However, all articles were evaluated to have findings of value and were not excluded based on the quality appraisal. The full quality appraisal is shown in Appendix S4.

### 3.2 Overview of Studies

Studies reported on care coordination (*n* = 5), patient or lay navigation (*n* = 5) and case management (*n* = 6) and used a variety of methodological approaches to examine implementation. Study designs were descriptive (*n* = 11), observational (*n* = 3) and experimental (*n* = 2). Studies were conducted in the USA (*n* = 9), Canada (*n* = 3), Australia (*n* = 1), Mexico (*n* = 1), Scotland (*n* = 1) and Singapore (*n* = 1). Studies were most often conducted in multiple sites (*n* = 9) and focused on populations with chronic conditions (*n* = 4), frequent use of hospital services (ED presentations, hospital admissions) (*n* = 3), stroke (*n* = 3), cancer (*n* = 2), substance use disorders (*n* = 1), mixed populations (*n* = 1), pregnant/post‐partum women (*n* = 1) and veterans with complex social needs (*n* = 1). Primary outcomes reported related to a mix of clinical and program outcomes (*n* = 2), experiences of care coordination (*n* = 2), evaluation of implementation (*n* = 2), facilitators and barriers to implementation (*n* = 2), feasibility (*n* = 2), fidelity (*n* = 1), cost and cost savings of the intervention (*n* = 1), adaptations over the course of implementation (*n* = 1), organisational readiness to implement change (*n* = 1), access to multidisciplinary support (*n* = 1) and characteristics of care coordination and their implementation contexts that help improve patient outcomes (*n* = 1). There was often a lack of clarity as to whether the studies were efficacy or effectiveness trials under “real‐world” hospital settings and appeared to be a hybrid of both. There was an even mix of studies evaluating pilot, experimental or feasibility interventions (*n* = 8) and real‐world implementation (*n* = 8). Further characteristics of included studies can be found in Table 1.

**TABLE 1:** Characteristics of included studies.

Study	Country	Primary aim	Study design	Setting	Study participants	Patient population	Sites	Chronic care condition	Type of care coordination program	Models, frameworks, theories	Measured outcomes
Blignault et al. (2021)	Australia	To explore patient, family and service provider experiences and views and to document and refine the model of care for Aboriginal adults with chronic conditions	Descriptive, qualitative study adopted a strengths‐based/appreciative enquiry approach	Hospital	Present and past ATOC staff members, relevant clinicians and managers, primary care and community service providers, and aboriginal patients and their families	Aboriginal adults admitted to hospital with a chronic condition or palliative care	2	Cardiovascular disease, diabetes, chronic obstructive pulmonary disease, chronic kidney disease (but not on dialysis) and asthma	Aboriginal Transfer of Care (care coordination)	*Ngaa‐bi‐nya* framework	Experiences and views of Aboriginal patients and their families/carers, ATOC team members and other hospital staff and community‐based service providers from government and non‐government organisations
Galarraga et al. (2021)	USA	To evaluate care coordination across an entire state's hospital system and gather unique insights on physician and staff experiences with ED care coordination	Descriptive, qualitative interview study	EDs from a range of geographical locations (inner city, suburban, rural) across the state of Maryland	ED physicians and care coordination staff	Those most targeted were high‐ED utilizers, substance abuse patients, uninsured patients, and congestive heart failure patients	18	Most common clinical diagnosis substance abuse, chronic heart failure, chronic pulmonary disease, dementia/geriatric functional decline, diabetes, low risk chest pain, pneumonia, pregnancy	Care coordination	NR	Describe the scope and variation of ED care coordination programs and perceptions of effective aspects of and barriers to ED care coordination, and the impact of Maryland's hospital global budget program
Gesell et al. (2019)	USA	To evaluate the first phase of implementation of a billable transitional care management for patients discharged home with stroke or TIA	Descriptive, process evaluation guided by RE‐AIM—using mixed methods within a cluster‐randomised pragmatic trial	Hospitals across the state of North Carolina	Hospital audits, hospital staff, community‐based services and patients	Patients aged 18 years or older, diagnosed with ischemic stroke, hemorrhagic stroke (excluding subdural or aneurysmal haemorrhage), or TIA, and were discharged home	20	Stroke or TIA	COMPASS‐TC (care coordination)	RE‐AIM	Reach, effectiveness, adoption, implementation and maintenance
Green et al. (2023)	USA	To identify facilitators of and barriers to the activities of postpartum patient navigators and their integration into the medical care team at an urban academic medical center as they navigate patients through 1 year postpartum	Descriptive, mixed methods analysis of implementation data within an ongoing RCT	Urban Academic Medical Centre	Patient navigator logs	Pregnant and postpartum individuals enrolled as an outpatient (after 30 weeks of gestation) or prior to hospital discharge following delivery—for low‐income individuals receiving Medicaid‐funded prenatal care	1	Pregnancy and post‐partum	Patient Navigation	C‐FIR	Facilitators and barriers to implementation
Horný et al. (2017)	USA	To improve glycemic control, increase patient engagement with the healthcare system, and improve the efficiency of care	Observational retrospective cohort study with pre‐ and post‐intervention periods	Outpatient clinic at an urban hospital	Patients	All patients enrolled in the diabetes clinic with A1c ≥ 8.5% and at least one appointment no‐show in the last year	1	Diabetes mellitus	Patient navigation	NR	Medical outcomes: Levels of A1C, LDL, cholesterol, triglycerides, random urine microalbumin Program outcomes: number and type of encounters, and scheduled appointment outcomes
Hudon et al. (2022)	Canada	To identify characteristics of case management programs, and the contexts where they are implemented, that help improve patient self‐management, experience of integrated care, and healthcare services use	Descriptive, multiple embedded case study with a mixed methods design	Hospital	Key informants involved in the six case management programs and healthcare services used by patients with complex health needs, and patients who were frequent users of hospital services (≥ 6 ED visits or ≥ 3 hospitalisations in the previous year)	Patients who were frequent users of hospital services (≥ 6 ED visits or ≥ 3 hospitalisations in the previous year)	6	NR	Case management	Chaudoir	Identification of characteristics of case management program and their contexts that can be related to the examined outcomes (e.g., patient self‐management, experience of integrated care, and health service use), and number of ED frequent users
Kahan et al. (2016)	Canada	To examine the acceptability of the multi‐organisational brief intervention using stakeholder perspectives, and identify barriers and facilitators of early implementation	Descriptive, qualitative evaluation study	Hospital	Stakeholders including direct service providers, managers and administrators from community mental health organisations, hospitals and Community Health Centres and other stakeholders from and members of the Frequent Users Advisory Committee	Adult frequent ED users (≥ 5 visits/year) with at least one visit for a mental health or substance use‐related concern	3	Mental health or substance use‐related as defined by ICD‐10 diagnostic codes	CATCH‐ED (brief case management)	Ecological framework and CFIR (informed development of interview guide)	Perceptions of program strengths and limitations, barriers and facilitators of early program implementation and perceived project acceptability, service delivery process (for frontline service providers)
Kimbell et al. (2018)	Scotland	To evaluate the feasibility of a nurse‐led, supportive care intervention for patients with advanced liver disease for whom liver transplantation was contraindicated	Descriptive, feasibility trial with a mixed‐method evaluation	Inpatient ward or outpatient clinic (referred in) at a tertiary hospital	Patients, carers, case linked care professionals e.g., GPs	Patients aged over 18 years with decompensated cirrhosis	1	Decompensated cirrhosis	Supportive care liver nurse specialist (case management)	NR	Feasibility of the intervention and evaluation, acceptability of the individual components of the intervention to patients, families and professionals, modifications to the intervention suggested by participants, and impact of the intervention
Lutz et al. (2020)	USA	To evaluate the implementation of COMPASS‐TC into complex real‐world settings was evaluated to identify successes and challenges with integration into the clinical workflow	Descriptive, process evaluation within a cluster‐randomised pragmatic trial	Hospitals in North Carolina	Hospital participation in surveys, group calls, group or individual interview with registered nurse post‐acute care coordinators, advanced practice providers including nurse practitioners, physician assistants and physicians, director of implementation, frontline clinicians, administrators	Patients aged 18 years or older, diagnosed with ischemic stroke, hemorrhagic stroke (excluding subdural or aneurysmal haemorrhage), or TIA and were discharged home	19	Stroke or TIA	COMPASS‐TC (care coordination)	RE‐AIM	Organisational readiness to implement change, adoption, reach, and implementation
Markle‐Reid et al. (2020)	Canada	To evaluate the feasibility of a larger RCT to examine the effectiveness of this integrated TC intervention for community‐living older adults with stroke and multimorbidity newly discharged from hospital and referred to an outpatient stroke rehabilitation setting	Observational prospective one‐group pre‐post pragmatic study	Outpatient clinic at a large urban academic teaching hospital	Patients	All older age (> 55 years), hospitalisation with a confirmed diagnosis of stroke, two or more comorbid chronic conditions	1	Stroke, comorbid conditions (NR)	Hospital‐to‐home transitional carestroke (care coordination)	Normalisation Process Theory (feasibility) ad Lorig and Holman's Self‐Management Theory (intervention)	Feasibility and effectiveness of implementing the intervention
McCreight et al. (2022)	USA	(1) Focus on the types, nature, and frequency of adaptations longitudinally; (2) discussing the strengths and limitations of different adaptation assessment methods; (3) presenting specifics about the use of process mapping to assess adaptations; and (4) recommending specific directions for future research and pragmatic use of adaptation methods	Descriptive, longitudinal multi‐method approach	VA Medical Centre (Rocky Mountain Region and Midwest Region)	Community transitions social worker, site champions, members of the Implementation and Evaluation team	Dual‐use Veterans who access both the Veterans Health Administration and non‐VA EDs	2	Have diverse and complex needs, address social determinants of health	Advanced Care Coordination program (case management)	RE‐AIM and the enhanced Expanded Framework for Reporting Adaptations and Modifications	Number and type and adaptations and how evolved over the course of implementation, if adaptations were planned, what was changed, why changed, who initiated change, impact of change
Nurjono et al. (2019)	Singapore	To evaluate the implementation fidelity of the National University Health System‐Regional Health System transitional care program to explain the outcomes of the program	Descriptive, realist evaluation, convergent parallel mixed‐methods study	Health system (tertiary, acute and community hospitals)	Patients and their families, health professionals	Elderly patients and frequent admitters (3 or more) with multiple chronic conditions, limited ambulation and caregiver(s) at home	1	NR	Post‐discharge care program (case management)	CFIF	Adherence to components of the program, frequency and duration (dose) of the enrolment, and moderating factors influencing the level of fidelity
Orme et al. (2022)	USA	To estimate the costs and cost savings of the NavSTAR intervention	Experimental, RCT with parallel design	Major urban medical centre and academic teaching hospital	Patients/health service staff	Hospitalised medical/surgical patients with comorbid substance use disorder	1	SUD	NavSTAR (patient navigation)	NR	Patient navigation and costs, healthcare data and costs, and cost savings
Rocque et al. (2016)	USA	To describe the development, infrastructure, lay navigation selection and training, and program operations of the Patient Care Connect Program	Descriptive	Health System Cancer Community Network cancer centres	NR	All Medicare primary fee‐for‐service beneficiaries ≥ 65 years old, with a cancer diagnosis after 2008, are eligible—special emphasis is placed on individuals with metastatic disease, cancers with high morbidity and mortality (e.g., lung, pancreatic), high distress, psychosocial needs or patients have barriers to receiving appropriate cancer care	12	Cancer	Patient Care Connect Program (lay navigation)	NR	Description of the implementation of navigation services including infrastructure development, physician engagement, navigator selection and training, navigation tools, patient identification strategies and monitoring of impact
Soto‐Perez‐de‐Celis et al. (2021)	Mexico	To evaluate if a patient navigation‐led multidisciplinary intervention could improve early access to multidisciplinary generalist supportive and palliative care, and lead io improvements in symptoms, quality of life, and engagement in advance care planning	Experimental, RCT	Oncology clinic at a large public hospital	Patients	Patients aged 18 years or older with metastatic solid tumours ≤ 6 weeks from diagnosis	1	Metastatic solid tumours	Patient navigation‐led supportive care	NR	Implementation of supporting and palliative care interventions defined as the proportion of patients who agreed to participate, complete initial assessment, met with the PN to discuss advance care planning, and obtained the recommended interventions within 12 weeks from enrolment Secondary—rate of implementation of each supportive and palliative care intervention; rate of AD completion; differences in QoL and supportive care needs between groups at 12 weeks
Wilcox et al. (2018)	USA	To determine the effectiveness in reducing 30‐day all‐cause readmissions for Medicare fee‐for‐service beneficiaries; the reach of the program with the target population; and the consistency in implementation of key program elements	Observational, retrospective study	Small academic hospital	Patients, hospital audits	Medicare beneficiaries hospitalised and aged 18 years or older	Evaluation from 1 site although implemented in 9 sites in this region	Congestive heart failure, myocardial infarction, stroke, TIA, pneumonia, chronic obstructive pulmonary disease, asthma, diabetes mellitus, renal disease (without haemodialysis), depressive symptoms, cognitive impairment	ComPass^2c^ (case management)	RE‐AIM	30‐day readmission rates, 7‐ and 14‐day post‐discharge physician follow‐up visits, 30‐day‐ED visits, observation visits, and mortality rates, and reach, effectiveness, implementation and patient activation

Abbreviations: A1C, Haemoglobin A1C; CATCH‐ED, Coordinating Access to Care from Hospital Emergency Departments; CFIF, Conceptual Framework for Implementation Fidelity; CFIR, Consolidated Framework for Implementation Research; COMPASS‐TC, Comprehensive Post‐Acute Stroke Services‐Transitional Care; ED, Emergency Department; ICD‐10, International Classification of Diseases 10th Revision; LDL, Low‐Density Lipoprotein; NavSTAR, Navigational Services to Avoid Rehospitalization; NR, Not Reported; RCT, Randomised Controlled Trial; RE‐AIM, Reach, Effectiveness, Adoption, Implementation, Maintenance; TC, Transitional Care; TIA, Transient Ischemic Attack; TOC, Aboriginal Transfer of Care; VA, Veterans Affairs.

### 3.3 Components of Case Management

The intervention implemented for each study was characterised by the standards of practice for case management (Table 2). One study could not be characterised according to the standards because a diverse range of interventions was implemented across the state of Maryland, USA (Galarraga et al. 2021). The case identification (screening) and assessment standard was well described by all studies (*n* = 15). However, risk stratification was less commonly identified (*n* = 4). For the planning standard, all studies implemented specific activities (care coordination), integrating health and social services, and supports to meet client needs (*n* = 15), and most developed a care plan in partnership with the client (*n* = 10). The monitoring standard was generally less well described. Although most studies included a description of monitoring to ensure care plans were implemented and monitoring of the resulting services provided (*n* = 10), fewer studies described management of transitions across the care continuum and preparing for exit from the program (*n* = 3), disengagement from the program (*n* = 5) or feedback from clients for evaluation (*n* = 6). Similarly, the evaluation and outcomes standard was variably described. Most provided an assessment of services provided (*n* = 12) and effectiveness in supporting the client (*n* = 11). Some studies described client satisfaction (*n* = 7) and training provided (*n* = 7) although few described the assessment of costs (*n* = 3). Although all studies evaluated implementation of care coordination interventions, it was unclear if the interventions were sustained once the evaluation was complete.

**TABLE 2:** Components of case management met by the included studies.

	Case identification			Planning		Monitoring				Evaluation and outcomes				
	Screening	Assessment	Risk stratification	Planning	Implementing	Monitoring	Transitioning	Disengagement	Feedback	Client satisfaction	Assessment costs	Assessment of services provided	Effectiveness in supporting the client	Training
Blignault et al. (2021)	+	−	+	+	+	−	−	−	+	+	−	+	+	−
Galarraga et al. (2021)	NA	NA	NA	NA	NA	NA	NA	NA	NA	NA	NA	NA	NA	NA
Gesell et al. (2019)	+	+	−	+	+	+	−	−	−	−	−	+	+	−
Green et al. (2023)	+	+	−	+^a^	+	−	+	+	−	−	−	+	−	+
Horný et al. (2017)	+	+	−	−	+	+	−	−	−	−	−	+	+	+
Hudon et al. (2022)	+	+	−	+	+	−	−	−	−	−	−	−	−	−
Kahan et al. (2016)	+	+	−	+	+	−	−	−	−	−	−	−	−	−
Kimbell et al. (2018)	+	+	−	+	+	+	−	+	+	+	−	+	+	+^b^
Lutz et al. (2020)	+	+	−	+	+	+	−	−	−	−	−	−	−	−
Markle‐Reid et al. (2020)	+	+	−	+	+	+	−	+	+	+	+	+	+	+
McCreight et al. (2022)	+	+	+	+^a^	+	−	+	+	+	+	+	+	+	+
Nurjono et al. (2019)	+	+	+	+	+	+	+	+	+	+	−	+	+	−
Orme et al. (2022)	+	+	−	−	+	+	−	−	+	+	+	+	+	−
Rocque et al. (2016)	+	+	+	−	+	+	−	−	−	+	−	+	+	+
Soto‐Perez‐de‐Celis et al. (2021)	+	+	−	+	+	+	−	−	−	−	−	+	+	−
Wilcox et al. (2018)	+	+	+	+	+	+	−	−	−	−	−	+	+	+

Abbreviations: −, Not Reported; +, Reported; NA, Not Applicable.

^a^ Unclear if developed in collaboration with client.

^b^ Training not specific to case management.

### 3.4 Consolidated Framework of Implementation Research Domains

A heat map depicting the frequencies of the CFIR constructs is presented in Table 3.

**TABLE 3:** Heat map depicting frequencies of the Consolidated Framework of Implementation Research constructs. [Colour table can be viewed at wileyonlinelibrary.com]

CFIR domain	CFIR construct	Blignault et al. (2021)	Galarraga et al. (2021)	Gesell et al. (2019)	Green et al. (2023)	Horný et al. (2017)	Hudon et al. (2022)	Kahan et al. (2016)	Kimbell et al. (2018)	Lutz et al. (2020)	Markle‐Reid et al. (2020)	McCreight et al. (2022)	Nurjono et al. (2019)	Orme et al. (2022)	Rocque et al. (2016)	Soto‐Perez‐de‐Celis et al. (2021)	Wilcox et al. (2018)
Innovation Domain	Innovation Source																
	Innovation Evidence‐Base																
	Innovation Relative Advantage																
	Innovation Adaptability																
	Innovation Trialability																
	Innovation Complexity																
	Innovation Design																
	Innovation Cost																
Outer Setting Domain	Critical Incidents																
	Local Attitudes																
	Local Conditions																
	Partnerships & Connections																
	Polices & Laws																
	Financing																
	External Pressure																
Inner Setting Domain	Physical Infrastructure																
	Information Technology Infrastructure																
	Work Infrastructure																
	Relational Connections																
	Communications																
	Human Equality‐Centredness																
	Recipient‐Centredness																
	Deliverer‐Centredness																
	Learning‐Centredness																
	Tension for Change																
	Compatibility																
	Relative Priority																
	Incentive Systems																
	Mission Alignment																
	Available Resources																
	Access to Knowledge & Information																
Individuals Domain	Roles Subdomain—High‐Level Leaders																
	Roles Subdomain—Mid‐Level Leaders																
	Roles Subdomain—Opinion Leaders																
	Roles Subdomain—Innovation Deliverers																
	Characteristics Subdomain—Need																
	Characteristics Subdomain—Capability																
	Characteristics Subdomain—Opportunity																
	Characteristics Subdomain—Motivation																
Implementation Process Domain	Teaming																
	Assessing—Innovation Deliverers																
	Assessing—Innovation Recipients																
	Assessing Context																
	Planning																
	Tailoring Strategies																
	Engaging—Innovation Deliverers																
	Engaging—Innovation Recipients																
	Doing																
	Reflecting & Evaluating—Implementation																
	Reflecting & Evaluating—Innovation																
	Adapting																

*Note:* Key: 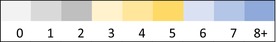.

#### 3.4.1 Domain 1: Innovation Domain

The innovation domain refers to the characteristics of the intervention (e.g., adaptability, trialability, complexity, cost). Although the innovation domain was not a strong focus of the included studies, constructs identified included complexity and cost. Innovation complexity was identified as challenging because of the need for variable components of the program to address patient heterogeneity, the interactions of numerous actors, the participant, care team members and community organisations with different perspectives (Nurjono et al. 2019; Green et al. 2023), the wide variability of tasks (Green et al. 2023), and detail of the program components (Nurjono et al. 2019). Cost was a relevant determinant lowering barriers for adoption and facilitating implementation in two important respects: the need for the intervention to be resource neutral, that is, use of existing staff and information systems to work in new ways (Nurjono et al. 2019; Blignault et al. 2021); and recognition that while intervention costs increased, non‐intervention costs decreased (e.g., admissions, ED visits, use of ambulance and 911 services) (Markle‐Reid et al. 2020; Orme et al. 2022).

#### 3.4.2 Domain 2: Outer Setting Domain

The outer setting domain relates to the economic, political, and social contexts outside the organisation in which the intervention is being implemented (e.g., local conditions, partnerships and connections, polices and laws). Common determinants included partnerships and connections, policies and laws, and financing.

The partnerships and connections between the inner (hospital) setting and the outer setting were important for successful implementation. Implementation of care coordination interventions created new partnerships (Galarraga et al. 2021; Blignault et al. 2021; Markle‐Reid et al. 2020; Rocque et al. 2016), strengthened existing partnerships between services (Blignault et al. 2021; Hudon et al. 2022) and built creative partnerships in rural communities (e.g., partnership with the local paramedicine program) (Lutz et al. 2020). Partnering with a local health authority enhanced priority access to primary care and mental health and addictions counselling (Kahan et al. 2016). Similarly, the development of a community resource network, where care coordinators worked with local‐community based organisations, helped to secure resources and facilitated referrals to local health and human services (Green et al. 2023; Lutz et al. 2020). Characteristics of strong partnerships were a shared purpose, understanding, joint leadership (Blignault et al. 2021), in‐kind contributions (Blignault et al. 2021; Kahan et al. 2016), multiple agency commitment (Kahan et al. 2016) and strong governance at the service delivery, organisational and system level (Blignault et al. 2021). Further, strong leadership by case managers also positively influenced the patient experience by facilitating collaboration among professionals and transitions between health services (Hudon et al. 2022). In contrast, with an absence of partnerships and connections, care coordination interventions were restricted by inadequate access to community‐based services (Galarraga et al. 2021; Nurjono et al. 2019), specialists with limited appointment availability (Galarraga et al. 2021; Kahan et al. 2016), lengthy wait lists for community mental health and addiction counselling (Kahan et al. 2016), and the inability to access patient information from community‐based services (Green et al. 2023). The decentralised structure of the health system in Canada meant navigating voluntary partnerships with many different organisations was complex (Kahan et al. 2016). Interpersonal connections and knowledge of community‐based services were also difficult to achieve (Green et al. 2023; Markle‐Reid et al. 2020; Hudon et al. 2022) and meant care remained siloed and fragmented, rather than integrated and coordinated (Nurjono et al. 2019; Hudon et al. 2022).

Policies and laws mediated the implementation of care coordination interventions. For example, at a community level, the adoption of the Global Budget Revenue (GBR) by the state of Maryland, USA, increased community services, encouraged hospitals to invest in strategies to improve community supports, and examined population health (Galarraga et al. 2021). At a patient level, the GBR encouraged shifting to holistic care, fostering a focus on implementing comprehensive care plans, care coordination and addressing social determinants (Galarraga et al. 2021). In contrast, where EDs and hospitals were pressured to reduce admissions, focus was placed on avoiding patient admissions, contributing to increased length of stay and wait times (Galarraga et al. 2021). This negatively impacted patient outcomes, including inappropriate discharge and patient satisfaction (Galarraga et al. 2021). Further, a conflict arose from declining ED activity between ED physicians being reimbursed on a fee‐for‐service basis versus hospital global budgets (Galarraga et al. 2021). Similarly, policy changes at a Quebec health system level resulted in the centralisation of health and social services and disruption of services (Hudon et al. 2022). This also disrupted the implementation of the case management program because of the instability caused by a lack of certainty surrounding sustainability and high staff turnover, with negative impacts on care integration (Hudon et al. 2022).

Financial support from external sources (e.g., government) facilitated implementation by covering staff costs, transport fees, training expenses and service costs of providing the program and increased patient adoption as patients paid minimal fees in a Singapore context (Nurjono et al. 2019). In contrast, while the GBR reforms increased care coordination services and staffing, there was insufficient financial support for staff coverage at different times (e.g., evening) (Galarraga et al. 2021), and barriers related to inadequate coverage for necessary services for uninsured and publicly insured patients, notably in a USA context (Galarraga et al. 2021; Lutz et al. 2020). There were also challenges to cover costs of implementation because of competition for reimbursement; for example, providers were unable to use the allocated billing code because the affiliated primary care providers were already claiming the only available billing code (Lutz et al. 2020).

#### 3.4.3 Domain 3: Inner Setting Domain

The inner setting domain refers to the complexity within the organisation influencing the performance of the inner setting where interventions are implemented (e.g., structural characteristics, relational connections, culture, access to knowledge and information). Work infrastructure, relational connections, culture, access to knowledge and information were commonly referred to as determinants.

A supportive work infrastructure was important for successful implementation and delivery of care coordination interventions. Multidisciplinary collaboration involving care coordinators facilitated the exchange of information (Galarraga et al. 2021; Blignault et al. 2021; Markle‐Reid et al. 2020) and supported the delivery of the program (Nurjono et al. 2019). Several studies also suggested collaborative multidisciplinary care with diverse clinical providers and distinct areas of expertise required clarification of the roles and responsibilities of each discipline (Galarraga et al. 2021; Nurjono et al. 2019; Blignault et al. 2021; Markle‐Reid et al. 2020; Rocque et al. 2016; Kahan et al. 2016; McCreight et al. 2022). Clarifying roles and communicating the roles of disciplines helped coordinate care, enabled appropriate referrals (Blignault et al. 2021; Markle‐Reid et al. 2020), identified who was responsible for follow‐up action (Blignault et al. 2021), and avoided duplication of services (McCreight et al. 2022). In contrast, unclear accountability led to variability in adherence to evidence‐based practice and inconsistency in implementation (Lutz et al. 2020; Kahan et al. 2016). Similarly, a lack of role clarity and specialised tasks associated with diverse professions contributed to difficulty providing holistic care and managing complex patients, causing delays in care (Nurjono et al. 2019). Implementation was also complicated by the wide variability of tasks and interactions required to address diverse patient needs (Green et al. 2023). Several studies noted the high turnover of staff (e.g., care coordinators, management level) (Hudon et al. 2022; Lutz et al. 2020; Kahan et al. 2016; Gesell et al. 2019) negatively impacted the openness of patients and providers, mixed program effects (Hudon et al. 2022), inconsistencies in implementation (Lutz et al. 2020), and missed finding and enrolling cases (Gesell et al. 2019). Further, busy work environments with large and rotating staff created challenges to promote program awareness and referrals (Kahan et al. 2016).

Relational connections were important, with several examples of formal and informal relationships evident, and the exchange of information a dominant feature of these relationships (Galarraga et al. 2021; Blignault et al. 2021; Markle‐Reid et al. 2020; Hudon et al. 2022; Kahan et al. 2016). Relationships with robust communication, information sharing, and exchange were important at the service delivery level (Blignault et al. 2021; Markle‐Reid et al. 2020; Hudon et al. 2022; Kahan et al. 2016; McCreight et al. 2022) facilitating the implementation and delivery of the program (Nurjono et al. 2019), improving care planning (Blignault et al. 2021; Markle‐Reid et al. 2020), care coordination (Green et al. 2023; Hudon et al. 2022), care delivery (Galarraga et al. 2021) and increased familiarity, and relationship‐building enhanced implementation (Green et al. 2023). Further, the onsite presence (Kahan et al. 2016) of a well‐known case manager located near the providers helped foster relationships and information sharing (Hudon et al. 2022). In contrast, the lack of information sharing between clinical, care coordination and outpatient care teams limited the effectiveness of patient care, with providers unaware of which services had already been provided (Galarraga et al. 2021). Care team members were also reluctant to integrate the care coordination role because of their unfamiliarity with the role (Green et al. 2023). Notably, large frequently rotating staff created challenges for program awareness and consumer referrals, in part due to the lack of ongoing communication with ED staff (Kahan et al. 2016).

Culture was a commonly cited determinant and refers to the shared values, beliefs, and norms related to human‐equality‐centredness (e.g., inherent value of each human as equal), recipient‐centredness (e.g., supporting and addressing the needs of individual patients), deliverer‐centredness (e.g., supporting and addressing the needs of deliverers) and learning‐centredness (e.g., continuous learning informed by use of data to inform change). Human equality‐centredness was challenging, notably in the USA context, with common barriers including affordability of care and a lack of or inadequate insurance (Galarraga et al. 2021; Lutz et al. 2020). Recipient centredness facilitated prioritising patient needs through review of patients prior to leaving hospital (Blignault et al. 2021), high adaptability and individualisation (Green et al. 2023), responding to patient‐identified goals and high accessibility to the program (e.g., re‐entry if needed) (Kahan et al. 2016). Further, connecting with patients disengaged from routine services resulted in more timely, planned and person‐centred hospital admissions (Kimbell et al. 2018). In contrast, in an Australian context, lack of cultural safety for Aboriginal people resulted in self‐discharge against medical advice (Blignault et al. 2021). Deliver‐centredness was not often prioritised, with insufficient staff and release time for the program, lack of training resulting in increased workload and extra administrative tasks (Galarraga et al. 2021; Nurjono et al. 2019; Markle‐Reid et al. 2020; Lutz et al. 2020), contributing to physician burnout (Galarraga et al. 2021) and lowered morale and responsiveness (Nurjono et al. 2019). Learning centredness facilitated implementation by encouraging collaboration and continual improvement informing organisational practices (Galarraga et al. 2021; Kahan et al. 2016).

The accessibility of guidance and training was important for implementation. For example, providing training in navigation and healthcare topics for lay navigators with limited prior healthcare experience facilitated implementation (Green et al. 2023). In contrast, inadequate, highly variable, or lack of training for care coordinators made it difficult to deliver the intervention and resulted in variations in delivery (Markle‐Reid et al. 2020; Hudon et al. 2022; Kahan et al. 2016), difficulties providing holistic care and management for patients with complex needs, and caused delays in care (Nurjono et al. 2019). Further, motivational interviewing was identified as a gap in training for lay navigators (Green et al. 2023) and lack of knowledge about community resources limited the ability to assist patients to navigate the healthcare system, that is, connect the patient to community resources (Galarraga et al. 2021; Markle‐Reid et al. 2020). While provision of protocols and manuals guided standardised delivery of the program and facilitated implementation (Nurjono et al. 2019; Markle‐Reid et al. 2020), the lack of well‐defined protocols led to improvisation to the components and dose of the program (Nurjono et al. 2019).

#### 3.4.4 Domain 4: Individuals Domain

The individuals domain relates to the roles (e.g., leaders, implementation leads, innovation deliverers) and characteristics (e.g., capability, opportunity) of individuals involved with the intervention and/or implementation process.

##### 3.4.4.1 Roles

The most identified implementation determinants were high‐level leaders, opinion leaders and innovation deliverers. Regarding high‐level leaders, many studies suggested leadership influenced the implementation of care coordination interventions. High‐level leaders were defined variously and across levels as senior managers, including case managers (Hudon et al. 2022; Lutz et al. 2020), the hospital administration and board (Lutz et al. 2020), the executive (Blignault et al. 2021) and the health authority (Hudon et al. 2022). Strong leadership and support enabled the implementation of the case management program to be maintained or resumed during the reform of the Quebec health system and enabled autonomy for case managers to adapt to the needs of patients and providers (Hudon et al. 2022) and develop creative local partnerships and address issues as they arose (Lutz et al. 2020). Changing leadership was noted to result in mixed program effects (Hudon et al. 2022) and successive adaptations throughout implementation (Nurjono et al. 2019).

Opinion leaders included ‘champions’ identified as standout hospital administrators or clinicians (Lutz et al. 2020) or medical directors (e.g., medical, radiation and surgical oncologists) with influence to secure multi‐level buy‐in and support for the intervention (e.g., with administrators, care team members involved in implementation and direct support staff) in a USA context (Rocque et al. 2016; Lutz et al. 2020).

Innovation deliverers are individuals who directly or indirectly deliver the innovation and include care coordinators (often a nurse or social worker), allied health and clinicians. Innovation deliverers who are well supported by their managers were able to create more personalised care trajectories, optimise patient transition through care pathways, and use services more appropriately (Hudon et al. 2022). Further, innovation deliverers were more satisfied and motivated when part of a team and when provided feedback about how their efforts were helping (Blignault et al. 2021). However, innovation deliverers isolated in their work suggested the need for a stronger team structure to facilitate personal and clinical support, information and resource sharing, and noted the lack of monitoring, support and supervision led to variation in the services provided (Kahan et al. 2016). Similarly, lack of supportive structures including program direction, protocols and a lack of training led to lowered morale and responsiveness to the program over time (Nurjono et al. 2019).

##### 3.4.4.2 Characteristics

Capability was an important characteristic and determinant for successful implementation and refers to competence, knowledge and skills related to the role of the care coordinator. Clinical and cultural experience to build relationships of trust and familiarity (Galarraga et al. 2021; Hudon et al. 2022), community knowledge (Green et al. 2023; Blignault et al. 2021; Lutz et al. 2020), understanding of patients' needs (Galarraga et al. 2021; Green et al. 2023; Blignault et al. 2021) and of the hospital or hospital system (Lutz et al. 2020) were critical implementation success factors. Further, stakeholder engagement skills reduced resistance to the program (Hudon et al. 2022). Appropriate and timely linkage to community services was limited by care coordinators lack of knowledge or unfamiliarity with community resources (Galarraga et al. 2021; Nurjono et al. 2019; Markle‐Reid et al. 2020; Lutz et al. 2020). Knowledge about the purpose and components of the intervention, and the importance of connections between patients, health‐care providers and hospital and community‐based services increased knowledge of best practices and appropriate community services (Markle‐Reid et al. 2020). In contrast, the lack of a well‐developed program direction and protocols led to improvisation of components and dose of the program and a lack of confidence in addressing and advocating for patients (Nurjono et al. 2019). Specific skills for care coordinators highlighted included leadership, coordination (Hudon et al. 2022), networking (Green et al. 2023; Hudon et al. 2022), communication (Rocque et al. 2016; Hudon et al. 2022), adaptability, advocacy (Green et al. 2023; Lutz et al. 2020), as well as being empathetic, respectful, warm, trustworthy and empowering (Rocque et al. 2016).

#### 3.4.5 Domian 5: Implementation Process Domain

The implementation process domain refers to the activities and strategies used to implement the intervention (e.g., teaming, engaging, reflecting and evaluating). The most referred to determinants included teaming, assessing context, engaging innovation recipients, reflecting and evaluating, and adapting.

Teaming (e.g., intentionally coordinating and collaborating to implement the intervention) was important for the implementation of care coordination interventions with several examples of building and working in teams. Teams were often built around the intervention and included care coordinators (often a nurse or social worker), Aboriginal Liaison Officers, allied health, clinicians, administration, management, and research team members. Teams brought together a clinical and culturally competent workforce (Galarraga et al. 2021; Green et al. 2023; Blignault et al. 2021; Kahan et al. 2016; McCreight et al. 2022) to collaborate (Nurjono et al. 2019; Green et al. 2023; Blignault et al. 2021; Kahan et al. 2016; McCreight et al. 2022), problem solve (Galarraga et al. 2021; Blignault et al. 2021), resolve implementation issues (Lutz et al. 2020), information share (Galarraga et al. 2021; Markle‐Reid et al. 2020), receive feedback (Blignault et al. 2021) and provide support (Rocque et al. 2016; Kahan et al. 2016). Goodwill and a shared sense of purpose were important for teaming and providers were more satisfied and motivated when part of a team (Blignault et al. 2021). However, one study suggested establishing a small team is insufficient and success is dependent on active participation by other hospital staff involved in patient care (Blignault et al. 2021). Some studies described several teams with distinct functions involved in implementation efforts (e.g., leadership teams, super utilizer committees, site teams, implementation teams) (Galarraga et al. 2021; Rocque et al. 2016) and communication lapses limited the efficacy of each party's efforts in patient care (Galarraga et al. 2021).

Engaging innovation recipients refers to attracting and encouraging recipients to serve on the implementation team and/or participate in the innovation. All the included data relates to innovation recipients receiving the intervention; there was a gap in evidence for innovation recipients serving on the implementation team. Site location influenced follow‐up care, with participants more willing to attend scheduled appointments at hospital than primary care clinics (Lutz et al. 2020), and higher volume and urban locations associated with a lower chance of scheduling clinic appointments (Gesell et al. 2019). Positive experiences during hospitalisation and discharge improved health service engagement and reduced self‐discharge (Blignault et al. 2021). Further, patients introduced to the program before discharge were more frequently reached (Gesell et al. 2019), successful at completing their 2‐day follow‐up call (Lutz et al. 2020) and follow‐up clinic visit than those notified post‐discharge by mail or phone (Lutz et al. 2020; Gesell et al. 2019). Successful engagement also led to increased supportive and palliative care recommendations implemented, advanced directives completed (Soto‐Perez‐de‐Celis et al. 2021), post‐discharge physician follow‐up (Wilcox et al. 2018), arrivals to scheduled appointments and decreased no shows to scheduled appointments (Horný et al. 2017). A variable range of enrolled patients received the intervention (Lutz et al. 2020) and the intervention was often tailored to suit patient needs (Green et al. 2023; Markle‐Reid et al. 2020; Rocque et al. 2016). Notably, patients often declined outpatient rehabilitation services (Markle‐Reid et al. 2020), refused to participate in the program (Nurjono et al. 2019), refused home visit (Soto‐Perez‐de‐Celis et al. 2021), did not engage in the program with multiple unsuccessful attempts at contact (Green et al. 2023; Lutz et al. 2020), or did not attend follow‐up clinic visit due to issues with insurance co‐pays in the USA context, transportation, seeing the visit as redundant or with no added value (Lutz et al. 2020), were self‐sufficient in facilitating their own care (Green et al. 2023), or preferred hospital care with outside care perceived as inferior (Nurjono et al. 2019). At least one study implemented several strategies to improve engagement with the innovation (e.g., communicating importance of visit prior to discharge, reminding patients of clinic visit) but did not report the effectiveness of the strategies (Lutz et al. 2020).

Reflecting and evaluating included the collection of information about the success of implementation or of the innovation to promote learning and improvements (e.g., continuous quality improvement). Successful implementation was underpinned by a continual improvement process to identify and address issues. Continual improvement was facilitated by a community feedback loop from patients, family members, healthcare providers and community programs (Galarraga et al. 2021) and tracking performance metrics (e.g., enrolment of eligible patients, receipt of 2‐day call, attendance at clinic visit) (Lutz et al. 2020) or quality control audits to monitor progress and share results (e.g., at a site and navigator level to monitor progress, trends and compliance with protocols) (Rocque et al. 2016). Communication at regular team meetings (Markle‐Reid et al. 2020), annual meetings and at local events (Rocque et al. 2016) was important to share program successes, challenges, and best practices to maximise efficiency and uptake of the program. Real‐time monitoring meant sites were able to anticipate or identify challenges and develop strategies to manage them effectively (Rocque et al. 2016; Lutz et al. 2020). For example, one implementation team worked to resolve issues related to start‐up (e.g., unfamiliarity with technology, insufficient time between training and start‐up) in the first wave of implementation so they did not occur in subsequent waves (Lutz et al. 2020).

Similarly, continuous data monitoring and feedback was critical for adjusting the innovation (Nurjono et al. 2019; Rocque et al. 2016; Lutz et al. 2020; Kimbell et al. 2018). For example, eligibility criteria (Kimbell et al. 2018) and late or incorrect referrals (Nurjono et al. 2019) adversely affected recruitment, which were quickly resolved by clinician‐recommended changes (Kimbell et al. 2018) or through continuous engagement with healthcare providers to clarify selection criteria (Nurjono et al. 2019). Further, continuous data monitoring and feedback demonstrated program value and fostered a culture of quality improvement (Rocque et al. 2016) and prompted the implementation of several strategies (e.g., improve clinic attendance, negotiation of longer appointment time slots for comprehensive assessment, development of algorithm for patient identification, tracking systems to follow patients through the steps of the intervention) to improve program uptake and fidelity (Lutz et al. 2020).

Adaptations were made to the enrolment criteria (Nurjono et al. 2019; Kimbell et al. 2018), recruitment strategies and roles of different providers (Nurjono et al. 2019) in response to overly strict criteria (e.g., insufficient numbers for enrolment) (Kimbell et al. 2018), leadership changes or healthcare provider experience (Nurjono et al. 2019). Further adaptations were made to the components and dose of the intervention in response to patient preferences (Markle‐Reid et al. 2020), patient needs (Green et al. 2023) or health provider experience (Nurjono et al. 2019). McCreight et al. (2022) specifically documented adaptations using the enhanced framework for reporting adaptations and reported most adaptations took place during early‐ and mid‐implementation, were planned, with most decisions to make adaptations made by the entire or most of the Implementation and Evaluation team (McCreight et al. 2022). Further, most adaptations were involved with program delivery, related to a core component of the intervention, or to extend a core component of the intervention (McCreight et al. 2022). Adaptations were made on the basis of practical considerations, in response to internal issues, with the intent to improve implementation, and perceived to impact the implementation dimension of the Reach, Effectiveness, Adoption, Implementation and Maintenance framework (a framework used to guide the planning and evaluation of programs) (McCreight et al. 2022).

## 4 Discussion

This review is the first to comprehensively evaluate the implementation of complex and holistic patient‐centred models of care coordination delivered within a hospital context. Despite increasing scientific awareness of implementation science, we only identified 16 studies that met the inclusion criteria, from a select group of countries, suggesting a lack of evidence on how implementation factors influence care coordination interventions. From the limited evidence, intra‐ and inter‐organisational connectivity facilitated by experienced care coordinators was essential for the implementation of high‐quality care coordination programs. This review also identified the importance of continual improvement to facilitate adaptation to address implementation issues and ensure ‘fit’ with patient and contextual factors. Studies were limited to the USA, Australia, Canada, Mexico, Scotland and Singapore, meaning there is much to be explored across international contexts and health systems.

Recent reviews have identified key program factors for case management (Hudon et al. 2017), care coordination (Albertson et al. 2022) and facilitators and barriers to the implementation of patient navigation interventions (Kokorelias et al. 2021). However, these reviews were narrow in scope of intervention terms (e.g., patient navigation only) given the ambiguity and overlap in the definition of variously named interventions and the role of the care coordinators. Further, all interventions were included irrespective of differences in the breadth and intensity of support offered (e.g., holistic programs addressing health and social needs versus programs addressing one aspect of care such as medication adherence). Similarly, interventions were delivered from a broad range of settings including the hospital, primary care and community‐based services. Given context is dynamic and influences variations in outcomes (Greenhalgh et al. 2004), attempts to understand implementation across such diversity may contribute to mixed knowledge regarding implementation. This review describes the evidence for complex patients during transitions of care, a key, high‐risk subgroup.

The care coordination interventions in this review were designed to facilitate patient care across a network of multidisciplinary, multisectoral, intra‐ and inter‐organisational professionals. Establishing and maintaining networks for effective care of complex, multimorbid patients within organisational settings is likely more challenging than for other patient groups given the larger and more heterogeneous range of health and social care touchpoints associated with their management (Gualandi et al. 2019; Solh Dost et al. 2024). Transitions between care settings increase the likelihood that information is lost, and care planning is fragmented (Gualandi et al. 2019; Solh Dost et al. 2024) leading to poorer outcomes (e.g., readmissions, adverse events, patient and provider satisfaction) (World Health Organization 2016). Similarly, interventions involving the identification of service needs in one setting (e.g., hospital) and linkage to specialised treatment in another (e.g., mental health care) are challenging to implement because they involve multiple stakeholders with different organisational cultures, structures and processes, priorities and approaches to collaboration (Aarons, Fettes, et al. 2014b). These challenges underpin the call for a paradigm shift to adopt a people‐centred and integrated healthcare service approach to ensure people receive a continuum of healthcare at different levels and sites within the health system and according to their needs (World Health Organisation 2015). Care coordination interventions, multidisciplinary working and linking organisations have all been identified as important strategies to integrated care (Hughes et al. 2020). Linking organisations using technological resources (e.g., developing integrated care pathways), partnerships (e.g., multiagency cooperation to address a common problem) and collective accountability (e.g., incentivising high‐value care for defined populations) are suggested to facilitate integrated healthcare systems (Hughes et al. 2020). Strategies to integrate care are also influenced by the broader economic, political and social contexts contributing to health system complexity (Greenhalgh et al. 2004), and highlight the importance of a combination of several strategies at multiple levels to facilitate connectivity between previously disconnected parts of the health system.

Care coordination interventions are a micro‐level example of integrated care, focusing on managing individual patients, and attempt to overcome meso‐ and macro‐level health system factors (Valentijn et al. 2013). The skill and experience of care coordinators are central to delivering high‐quality care coordination, working within multidisciplinary teams, crossing disciplinary boundaries, coordinating care across multiple services and linking organisations. This review showed experienced care coordinators with high‐level networking and communication skills and knowledge of community resources were essential to build intra‐ and inter‐organisational relationships to create a network of care around people. In contrast, a high turnover of care coordinator staff and a lack of supportive organisational structures, including leadership, knowledge of community services, and training, negatively influenced implementation. The role of care coordinators can be conceptualised as multi‐faceted and complex, with the development of key relationships and networks central to their role in problem solving, spanning system boundaries, and brokering multidisciplinary, multi‐system support (Hannan‐Jones et al. 2021). Studies have shown that skilled care coordinators and support and training of care coordinators facilitate implementation (Hudon, Bisson, et al. 2023b; Hudon, Chouinard, et al. 2023a) while insufficient training and poor collaboration with other health‐care providers are barriers to implementation (Joo and Huber 2018). However, the reliance on the ability of individual care coordinators to overcome system factors may not be sustainable or scalable and overlooks broader context and structural barriers. The development of a broader team of people with skills across a range of roles (e.g., leadership, champions, opinion leaders) and creating opportunities for shared learning and knowledge exchange (e.g., to break down interorganisational barriers) may build capacity to support care coordinators (Macfarlane et al. 2011). Similarly, alignment across multiple levels of leadership across systems and organisations may help to further develop a context that supports the role of care coordinators and facilitates implementation of care coordination interventions (Aarons, Ehrhart, et al. 2014a). A combination of approaches may be necessary to compensate for variations in individual experience and support care coordinators to work fluently across health and social settings.

Care coordination interventions are rarely able to be implemented as a pre‐defined, ‘generic’ model and often adaptation is needed to address local barriers to implementation and sustainability (e.g., changes in laws and policies, organisational priorities and governance, financing arrangements, healthcare team composition or patient cohort changes). Monitoring and evaluation are important strategies to facilitate effective implementation of care coordination interventions and to guide and inform adaptation (Chambers et al. 2013). This review suggests that successful implementation of care coordination interventions was facilitated by a continual improvement process (e.g., reflection and evaluation) to identify and address implementation issues. It is well recognised that adaptation is essential in real‐world contexts. For example, the Dynamic Sustainability Framework emphasises constant unpredictable change in a multi‐level context, the intervention itself, the organisation in which the intervention is delivered and the broader health system in which the organisation operates (Chambers et al. 2013). This suggests continuous and iterative adaptation is critical for sustainability and continued alignment between the intervention and organisational needs and capabilities (Chambers et al. 2013; Côté‐Boileau et al. 2019). This highlights the importance of organisational learning through use of information gathered in real‐time to allow for local tailoring (i.e., adaptation), to address barriers and increase fit with patient and contextual factors to successfully implement and sustain care coordination interventions.

This review highlighted tensions between standardising both the model and implementation of care coordination interventions. Complex interventions, such as care coordination, comprise multiple components and interact with a dynamic complex health system interconnected and interrelated across multiple layers and dimensions. The findings suggest that care coordination interventions were not one phenomenon; rather, they covered a range of conceptual models with different aims, activities, patient groups, and outcomes that meant different things to different people (e.g., patients, health professionals, organisations). Further, the included studies tended to adopt a broad evaluation lens without having a clear taxonomy to describe the intervention, the context in which the intervention was being implemented, or implementation processes effecting critical appraisal and reproducibility. For example, few articles described care coordination interventions, the implementation context, or the process of implementation in depth. Further, most studies rarely described if interventions were modified or adapted over time or if implementation strategies were used to address barriers to implementation. Although care coordination interventions are contextually shaped with commonalities, it is difficult to fully account for the nuance and complexity of different contexts and implementation processes to achieve an ‘ideal’ standard model ready to be implemented. Future studies should provide clear descriptions of the care coordination intervention using standardised taxonomies (e.g., case management), the care coordinator role (e.g., role clarity, experience, training and supporting structures) and the macro‐context (e.g., policy, funding, partnerships) into which the intervention is being implemented. There is also a need to test implementation strategies to leverage enablers and address barriers identified in this review, identify and determine the impact of adaptation, and the long‐term sustainability of care coordination interventions.

### 4.1 Strengths and Limitations

A strength of this review is the use of rigorous and robust methods to identify, appraise, and synthesise the literature related to care coordination interventions. It also uses CFIR, a practical framework to guide systematic assessment of potential barriers and facilitators that influence implementation efforts and aids the transferability and comparability of findings from this review to other implementation studies. A limitation of this review is that, given the considerable importance of context to successfully implement care coordination interventions, the 16 eligible studies likely do not represent a complete profile of issues experienced in practice. Similarly, insufficient descriptions of care coordination interventions and the context into which they were implemented made it difficult to draw conclusions and limited the generalisability of findings. While it is possible that some eligible studies were missed because of multiple and inconsistent terms used to describe both implementation and care coordination interventions, the review purposefully adopted a broad conceptual base to reduce the likelihood. Papers written in languages other than English were excluded, and as most of the included studies were conducted in developed countries, the results may be less relevant to developing nations.

## 5 Conclusions

This review demonstrates that care coordination interventions are complex and heterogenous, and that implementation is multi‐faceted. Studies have only been conducted in a small number of countries and contexts, leaving much more to be understood. Evidence from this review shows the importance of intra‐ and inter‐organisational relationships to connect previously disconnected parts of the health system, the skills and responsibilities of care coordinators to coordinate care across multiple services, and organisational learning to increase fit with contextual factors to support implementation. The review also suggests that diverse care coordination interventions and contextual factors make it difficult to describe a single explanatory model. More high‐quality studies are needed to identify commonalities and provide generalisable principles and characteristics associated with high performance, with broader geographical and health system coverage.

## Author Contributions

Conceptualisation: M.M., K.M.N., A.W.S., L.A. Data acquisition: M.M., S.W., M.M., H.B. Formal Analysis and interpretation: M.M., K.M.N., A.W.S. Original draft: M.M. Review and editing: S.W., M.M., H.B., V.L.V., L.A., A.W.S., K.M.N. Supervision: K.M.N., A.W.S., L.A., V.L.V.

## Conflicts of Interest Statement

The authors have declared no conflicts of interest.

## Supporting information


**Appendix S1:** jocn70102‐sup‐0001‐AppendixS1.docx.

**Appendix S2:** jocn70102‐sup‐0002‐AppendixS2.docx.

**Appendix S3:** jocn70102‐sup‐0002‐AppendixS3.docx.

**Appendix S4:** jocn70102‐sup‐0003‐AppendixS4.docx.

## Data Availability

The authors have nothing to report.
