# Supplementary material for: Implementation Outcomes and Their Determinants for Hospital‐Led Care Coordination Interventions Targeting Patients With Complex Care Needs: A Qualitative Systematic Review
Source: J Clin Nurs. 2025 Sep 15;35(3):1079–103. doi: 10.1111/jocn.70102 (PMC12862520; doi:10.1111/jocn.70102)
Supplement: Supplementary file 2 — Appendix S2: jocn70102‐sup‐0002‐AppendixS2.docx. [file JOCN-35-1079-s001.docx]

MEDLINE Complete (EBSCOhost)

| SEARCH ID# | SEARCH TERMS |
| --- | --- |
| S1 | TI ( (patient OR nurse OR peer OR community OR system OR care OR lay) n3 navigat* ) OR AB ( (patient OR nurse OR peer OR community OR system OR care OR lay) n3 navigat* ) |
| S2 | TI "ca?e manag*" OR AB "ca?e manag*" |
| S3 | TI ( (integrat* OR collaborat* OR coordinat* OR multidisciplin* OR interdisciplin* OR linkage) N3 (heath* OR care) ) OR AB ( (integrat* OR collaborat* OR coordinat* OR multidisciplin* OR interdisciplin* OR linkage) N3 (heath* OR care) ) |
| S4 | (MH "Patient Navigation") |
| S5 | (MH "Case Management") |
| S6 | (MH "Case Managers") |
| S7 | (MH "Delivery of Health Care, Integrated") |
| S8 | (MH "Patient Care Management") |
| S9 | (MH "Patient Care Team") |
| S10 | S1 OR S2 OR S3 OR S4 OR S5 OR S6 OR S7 OR S8 OR S9 |
| S11 | TI "secondary care" OR AB "secondary care" |
| S12 | TI "secondary health care" OR AB "secondary health care" |
| S13 | TI "secondary healthcare" OR AB "secondary healthcare" |
| S14 | TI "tertiary care" OR AB "tertiary care" |
| S15 | TI "tertiary health care" OR AB "tertiary health care" |
| S16 | TI "tertiary healthcare" OR AB "tertiary healthcare" |
| S17 | TI hospital* OR AB hospital* |
| S18 | TI emergency n2 service* OR AB emergency n2 service* |
| S19 | TI "accident and emergency" OR AB "accident and emergency" |
| S20 | TI casualty OR AB casualty |
| S21 | TI acute N3 (care OR setting) OR AB acute N3 (care OR setting) |
| S22 | TI "emergency department*" OR AB "emergency department*" |
| S23 | TI "emergency unit*" OR AB "emergency unit*" |
| S24 | TI "emergency ward*" OR AB "emergency ward*" |
| S25 | TI "emergency room*" OR AB "emergency room*" |
| S26 | TI ward* OR AB ward* |
| S27 | TI outpatient* OR AB outpatient* |
| S28 | TI inpatient* OR AB inpatient* |
| S29 | (MH "Hospitals+") |
| S30 | (MH "Tertiary Healthcare") |
| S31 | (MH "Outpatients") |
| S32 | (MH "Inpatients") |
| S33 | (MH "Hospital Departments+") |
| S34 | S11 OR S12 OR S13 OR S14 OR S15 OR S16 OR S17 OR S18 OR S19 OR S20 OR S21 OR S22 OR S23 OR S24 OR S25 OR S26 OR S27 OR S28 OR S29 OR S30 OR S31 OR S32 OR S33 |
| S35 | TI "knowledge trans*" OR AB "knowledge trans*" |
| S36 | TI "implementation science" OR AB "implementation science" |
| S37 | TI implement* N2 (evaluat* OR analysis OR outcome* OR process) OR AB implement* N2 (evaluat* OR analysis OR outcome* OR process) |
| S38 | TI "program implement*" OR AB "program implement*" |
| S39 | TI "process evaluation" OR AB "process evaluation" |
| S40 | TI "program development" OR AB "program development" |
| S41 | TI "program structure*" OR AB "program structure*" |
| S42 | TI "health services research" OR AB "health services research" |
| S43 | TI (program* OR evidence-based OR innovation* OR intervention*) N3 (integrat* OR adopt* OR routin* OR embed* OR sustain* OR fidelity OR uptake OR maintain* OR disseminat* OR diffus* OR accept* OR adopt* OR adher*) OR AB (program* OR evidence-based OR innovation* OR intervention*) N3 (integrat* OR adopt* OR routin* OR embed* OR sustain* OR fidelity OR uptake OR maintain* OR disseminat* OR diffus* OR accept* OR adopt* OR adher*) |
| S44 | TI "organi?ational change" OR AB "organi?ational change" |
| S45 | TI "change management" OR AB "change management" |
| S46 | TI "capacity building" OR AB "capacity building" |
| S47 | TI "scal* up" OR AB "scal* up" |
| S48 | TI "roll* out" OR AB "roll* out" |
| S49 | (MH "Diffusion of Innovation+") |
| S50 | (MH "Program Evaluation") |
| S51 | (MH "Program Development") |
| S52 | (MH "Process Assessment, Health Care") |
| S53 | (MH "Nursing Evaluation Research") |
| S54 | (MH "Organizational Innovation+") |
| S55 | (MH "Capacity Building") |
| S56 | S35 OR S36 OR S37 OR S38 OR S39 OR S40 OR S41 OR S42 OR S43 OR S44 OR S45 OR S46 OR S47 OR S48 OR S49 OR S50 OR S51 OR S52 OR S53 OR S54 OR S55 |
| S57 | S10 AND S34 AND S56 |
| S58 | LA English |
| S59 | DT 20130101-20221231 |
| S60 | S57 AND S58 AND S59 |

CINHAL Complete (EBSCOhost)

| SEARCH ID# | SEARCH TERMS |
| --- | --- |
| S1 | TI ( (patient OR nurse OR peer OR community OR system OR care OR lay) n3 navigat* ) OR AB ( (patient OR nurse OR peer OR community OR system OR care OR lay) n3 navigat* ) |
| S2 | TI "ca?e manag*" OR AB "ca?e manag*" |
| S3 | TI ( (integrat* OR collaborat* OR coordinat* OR multidisciplin* OR interdisciplin* OR linkage) N3 (heath* OR care) ) OR AB ( (integrat* OR collaborat* OR coordinat* OR multidisciplin* OR interdisciplin* OR linkage) N3 (heath* OR care) ) |
| S4 | (MH "Patient Navigation") |
| S5 | (MH "Case Management") |
| S6 | (MH "Case Managers") |
| S7 | (MH "Delivery of Health Care, Integrated") |
| S8 | (MH "Patient Care Plans") |
| S9 | (MH "Multidisciplinary Care Team") |
| S10 | S1 OR S2 OR S3 OR S4 OR S5 OR S6 OR S7 OR S8 OR S9 |
| S11 | TI "secondary care" OR AB "secondary care" |
| S12 | TI "secondary health care" OR AB "secondary health care" |
| S13 | TI "secondary healthcare" OR AB "secondary healthcare" |
| S14 | TI "tertiary care" OR AB "tertiary care" |
| S15 | TI "tertiary health care" OR AB "tertiary health care" |
| S16 | TI "tertiary healthcare" OR AB "tertiary healthcare" |
| S17 | TI hospital* OR AB hospital* |
| S18 | TI emergency n2 service* OR AB emergency n2 service* |
| S19 | TI "accident and emergency" OR AB "accident and emergency" |
| S20 | TI casualty OR AB casualty |
| S21 | TI acute N3 (care OR setting) OR AB acute N3 (care OR setting) |
| S22 | TI "emergency department*" OR AB "emergency department*" |
| S23 | TI "emergency unit*" OR AB "emergency unit*" |
| S24 | TI "emergency ward*" OR AB "emergency ward*" |
| S25 | TI "emergency room*" OR AB "emergency room*" |
| S26 | TI ward* OR AB ward* |
| S27 | TI outpatient* OR AB outpatient* |
| S28 | TI inpatient* OR AB inpatient* |
| S29 | (MH "Hospitals+") |
| S30 | (MH "Tertiary Health Care") |
| S31 | (MH "Emergency Service+") |
| S32 | (MH "Outpatient Service") |
| S33 | (MH "Outpatients") |
| S34 | (MH "Inpatients") |
| S35 | (MH "Secondary Health Care") |
| S36 | S11 OR S12 OR S13 OR S14 OR S15 OR S16 OR S17 OR S18 OR S19 OR S20 OR S21 OR S22 OR S23 OR S24 OR S25 OR S26 OR S27 OR S28 OR S29 OR S30 OR S31 OR S32 OR S33 OR S34 OR S35 |
| S37 | TI "knowledge trans*" OR AB "knowledge trans*" |
| S38 | TI "implementation science" OR AB "implementation science" |
| S39 | TI implement* N2 (evaluat* OR analysis OR outcome* OR process) OR AB implement* N2 (evaluat* OR analysis OR outcome* OR process) |
| S40 | TI "program implement*" OR AB "program implement*" |
| S41 | TI "process evaluation" OR AB "process evaluation" |
| S42 | TI "program development" OR AB "program development" |
| S43 | TI "program structure*" OR AB "program structure*" |
| S44 | TI "health services research" OR AB "health services research" |
| S45 | TI (program* OR evidence-based OR innovation* OR intervention*) N3 (integrat* OR adopt* OR routin* OR embed* OR sustain* OR fidelity OR uptake OR maintain* OR disseminat* OR diffus* OR accept* OR adopt* OR adher*) OR AB (program* OR evidence-based OR innovation* OR intervention*) N3 (integrat* OR adopt* OR routin* OR embed* OR sustain* OR fidelity OR uptake OR maintain* OR disseminat* OR diffus* OR accept* OR adopt* OR adher*) |
| S46 | TI "organi?ational change" OR AB "organi?ational change" |
| S47 | TI "change management" OR AB "change management" |
| S48 | TI "capacity building" OR AB "capacity building" |
| S49 | TI "scal* up" OR AB "scal* up" |
| S50 | TI "roll* out" OR AB "roll* out" |
| S51 | (MH "Diffusion of Innovation+") |
| S52 | (MH "Program Development+") |
| S53 | (MH "Process Assessment (Health Care)+") |
| S54 | (MH "Outcome Assessment") |
| S55 | (MH "Organizational Change") |
| S56 | (MH "Change Management") |
| S57 | (MH "Evaluation Research+") |
| S58 | S37 OR S38 OR S39 OR S40 OR S41 OR S42 OR S43 OR S44 OR S45 OR S46 OR S47 OR S48 OR S49 OR S50 OR S51 OR S52 OR S53 OR S54 OR S55 OR S56 OR S57 |
| S59 | S10 AND S36 AND S58 |
| S60 | LA English |
| S61 | DT 20130101-20221231 |
| S62 | S59 AND S60 AND S61 |

PsychINFO (EBSCOhost)

| SEARCH ID# | SEARCH TERMS |
| --- | --- |
| S1 | TI ( (patient OR nurse OR peer OR community OR system OR care OR lay) n3 navigat* ) OR AB ( (patient OR nurse OR peer OR community OR system OR care OR lay) n3 navigat* ) |
| S2 | TI "ca?e manag*" OR AB "ca?e manag*" |
| S3 | TI ( (integrat* OR collaborat* OR coordinat* OR multidisciplin* OR interdisciplin* OR linkage) N3 (heath* OR care) ) OR AB ( (integrat* OR collaborat* OR coordinat* OR multidisciplin* OR interdisciplin* OR linkage) N3 (heath* OR care) ) |
| S4 | DE "Case Management" |
| S5 | DE "Integrated Services" |
| S6 | S1 OR S2 OR S3 OR S4 OR S5 |
| S7 | TI "secondary care" OR AB "secondary care" |
| S8 | TI "secondary health care" OR AB "secondary health care" |
| S9 | TI "secondary healthcare" OR AB "secondary healthcare" |
| S10 | TI "tertiary care" OR AB "tertiary care" |
| S11 | TI "tertiary health care" OR AB "tertiary health care" |
| S12 | TI "tertiary healthcare" OR AB "tertiary healthcare" |
| S13 | TI hospital* OR AB hospital* |
| S14 | TI emergency n2 service* OR AB emergency n2 service* |
| S15 | TI "accident and emergency" OR AB "accident and emergency" |
| S16 | TI casualty OR AB casualty |
| S17 | TI acute N3 (care OR setting) OR AB acute N3 (care OR setting) |
| S18 | TI "emergency department*" OR AB "emergency department*" |
| S19 | TI "emergency unit*" OR AB "emergency unit*" |
| S20 | TI "emergency ward*" OR AB "emergency ward*" |
| S21 | TI "emergency room*" OR AB "emergency room*" |
| S22 | TI ward* OR AB ward* |
| S23 | TI outpatient* OR AB outpatient* |
| S24 | TI inpatient* OR AB inpatient* |
| S25 | DE "Hospitals" |
| S26 | DE "Emergency Services" |
| S27 | DE "Hospital Programs" |
| S28 | DE "Outpatients" |
| S29 | DE "Mental Health Services" |
| S30 | S7 OR S8 OR S9 OR S10 OR S11 OR S12 OR S13 OR S14 OR S15 OR S16 OR S17 OR S18 OR S19 OR S20 OR S21 OR S22 OR S23 OR S24 OR S25 OR S26 OR S27 OR S28 OR S29 |
| S31 | TI "knowledge trans*" OR AB "knowledge trans*" |
| S32 | TI "implementation science" OR AB "implementation science" |
| S33 | TI implement* N2 (evaluat* OR analysis OR outcome* OR process) OR AB implement* N2 (evaluat* OR analysis OR outcome* OR process) |
| S34 | TI "program implement*" OR AB "program implement*" |
| S35 | TI "process evaluation" OR AB "process evaluation" |
| S36 | TI "program development" OR AB "program development" |
| S37 | TI "program structure*" OR AB "program structure*" |
| S38 | TI "health services research" OR AB "health services research" |
| S39 | TI (program* OR evidence-based OR innovation* OR intervention*) N3 (integrat* OR adopt* OR routin* OR embed* OR sustain* OR fidelity OR uptake OR maintain* OR disseminat* OR diffus* OR accept* OR adopt* OR adher*) OR AB (program* OR evidence-based OR innovation* OR intervention*) N3 (integrat* OR adopt* OR routin* OR embed* OR sustain* OR fidelity OR uptake OR maintain* OR disseminat* OR diffus* OR accept* OR adopt* OR adher*) |
| S40 | TI "organi?ational change" OR AB "organi?ational change" |
| S41 | TI "change management" OR AB "change management" |
| S42 | TI "capacity building" OR AB "capacity building" |
| S43 | TI "scal* up" OR AB "scal* up" |
| S44 | TI "roll* out" OR AB "roll* out" |
| S45 | DE "Program Evaluation" |
| S46 | DE "Program Development" |
| S47 | DE "Organizational Change" |
| S48 | DE "Change Strategies" |
| S49 | DE "Innovation" |
| S50 | DE "Patient Reported Outcome Measures" |
| S51 | S31 OR S32 OR S33 OR S34 OR S35 OR S36 OR S37 OR S38 OR S39 OR S40 OR S41 OR S42 OR S43 OR S44 OR S45 OR S46 OR S47 OR S48 OR S49 OR S50 |
| S52 | S6 AND S30 AND S51 |
| S53 | LA English |
| S54 | DT 20130101-20221231 |
| S55 | S52 AND S53 AND S54 |

Embase (Ovid)

| SEARCH ID# | SEARCH TERMS |
| --- | --- |
| #1 | ((patient OR nurse OR peer OR community OR system OR care OR lay) NEAR/3 navigat*):ab,ti |
| #2 | 'ca?e manag*':ab,ti |
| #3 | ((integrat* OR collaborat* OR coordinat* OR multidisciplin* OR interdisciplin* OR linkage) NEAR/3 (heath* OR care)):ab,ti |
| #4 | 'case management'/de |
| #5 | 'case manager'/de |
| #6 | 'collaborative care team'/de |
| #7 | 'patient navigator'/de |
| #8 | 'patient navigator program'/de |
| #9 | 'care coordination'/de |
| #10 | 'care coordinator'/de |
| #11 | 'nurse navigator'/de |
| #12 | 'lay health worker'/de |
| #13 | 'care manager'/de |
| #14 | 'integrated care'/de |
| #15 | 'integrated care pathway'/de |
| #16 | 'linkage to care'/de |
| #17 | 'patient care planning'/de |
| #18 | #1 OR #2 OR #3 OR #4 OR #5 OR #6 OR #7 OR #8 OR #9 OR #10 OR #11 OR #12 OR #13 OR #14 OR #15 OR #16 OR #17 |
| #19 | 'secondary care':ab,ti |
| #20 | 'secondary health care':ab,ti |
| #21 | 'secondary healthcare':ab,ti |
| #22 | 'tertiary care':ab,ti |
| #23 | 'tertiary health care':ab,ti |
| #24 | 'tertiary healthcare':ab,ti |
| #25 | hospital*:ab,ti |
| #26 | (emergency NEAR/2 service*):ab,ti |
| #27 | 'accident and emergency':ab,ti |
| #28 | casualty:ab,ti |
| #29 | (acute NEAR/3 (care OR setting)):ab,ti |
| #30 | 'emergency department*':ab,ti |
| #31 | 'emergency unit*':ab,ti |
| #32 | 'emergency ward*':ab,ti |
| #33 | 'emergency room*':ab,ti |
| #34 | ward*:ab,ti |
| #35 | outpatient*:ab,ti |
| #36 | inpatient*:ab,ti |
| #37 | 'hospital'/exp |
| #38 | 'outpatient'/de |
| #39 | 'outpatient care'/de |
| #40 | 'inpatient care'/de |
| #41 | 'emergency health service'/exp |
| #42 | 'acute care hospital'/de |
| #43 | 'tertiary health care'/exp |
| #44 | 'secondary health care'/exp |
| #45 | #19 OR #20 OR #21 OR #22 OR #23 OR #24 OR #25 OR #26 OR #27 OR #28 OR #29 OR #30 OR #31 OR #32 OR #33 OR #34 OR #35 OR #36 OR #37 OR #38 OR #39 OR #40 OR #41 OR #42 OR #43 OR #44 |
| #46 | 'knowledge trans*':ab,ti |
| #47 | 'implementation science':ab,ti |
| #48 | (implement* NEAR/2 (evaluat* OR analysis OR outcome* OR process)):ab,ti |
| #49 | 'program implement*':ab,ti |
| #50 | 'process evaluation':ab,ti |
| #51 | 'program development':ab,ti |
| #52 | 'program structure*':ab,ti |
| #53 | 'health services research':ab,ti |
| #54 | ((program* OR 'evidence based' OR innovation* OR intervention*) NEAR/3 (integrat* OR adopt* OR routin* OR embed* OR sustain* OR fidelity OR uptake OR maintain* OR disseminat* OR diffus* OR accept* OR adopt* OR adher*)):ab,ti |
| #55 | 'organi?ational change':ab,ti |
| #56 | 'change management':ab,ti |
| #57 | 'capacity building':ab,ti |
| #58 | 'scal* up':ab,ti |
| #59 | 'roll* out':ab,ti |
| #60 | 'implementation science'/de |
| #61 | 'implementation'/de |
| #62 | 'implementation intention'/de |
| #63 | 'knowledge translation'/de |
| #64 | 'knowledge transfer'/de |
| #65 | 'fidelity'/de |
| #66 | 'fidelity level'/de |
| #67 | 'health services research'/de |
| #68 | 'change management'/de |
| #69 | 'translational research'/de |
| #70 | 'capacity building'/de |
| #71 | 'scale up'/de |
| #72 | 'innovation'/de |
| #73 | 'diffusion of innovation'/de |
| #74 | 'program evaluation'/exp |
| #75 | 'program development'/de |
| #76 | 'nursing evaluation research'/de |
| #77 | 'total quality management'/de |
| #78 | #46 OR #47 OR #48 OR #49 OR #50 OR #51 OR #52 OR #53 OR #54 OR #55 OR #56 OR #57 OR #58 OR #59 OR #60 OR #61 OR #62 OR #63 OR #64 OR #65 OR #66 OR #67 OR #68 OR #69 OR #70 OR #71 OR #72 OR #73 OR #74 OR #75 OR #76 OR #77 |
| #79 | #18 AND #45 AND #78 |
| #80 | [english]/lim |
| #81 | [2013-2022]/py |
| #82 | #79 AND #80 AND #81 |
