# Supplementary material for: Implementation Outcomes and Their Determinants for Hospital‐Led Care Coordination Interventions Targeting Patients With Complex Care Needs: A Qualitative Systematic Review
Source: J Clin Nurs. 2025 Sep 15;35(3):1079–103. doi: 10.1111/jocn.70102 (PMC12862520; doi:10.1111/jocn.70102)
Supplement: Supplementary file 3 — Appendix S3: jocn70102‐sup‐0002‐AppendixS3.docx. [file JOCN-35-1079-s003.docx]

| **Standard** | **Phases** | **Operationalisation for Systematic Review** |
| --- | --- | --- |
| Case identification (screening) and assessment | Screening | Screening of information about perceived client needs to evaluate appropriateness within the aims and objectives of the case management program, and offering the program through outreach or referral |
|  | Assessment | Ongoing collection and assessment of data from various sources (interviews, risk assessments) to identify client needs and underpins subsequent actions |
|  | Risk stratification | Determining the appropriate level of action based on the client’s situation, needs and interests |
| Planning (may include but is not limited to) | Planning | Develop a care plan in partnership with the client (establish objectives, goals, and interventions necessary) to meet a client’s needs |
|  | Implementing (care coordination) | Execution of the specific activities and interventions necessary for achieving the agreed goals within the care plan, including to organise, secure and integrate health and social services, and supports to meet needs |
| Monitoring (may include but is not limited to) | Monitoring | Monitoring to ensure the client’s care plan is being implemented and monitoring the quality of services being provided |
|  | Transitioning | A focus on moving the client across the care continuum in response to the client’s achievement towards goals within the care plan and preparing the client to exit the program or transfer to another program or setting |
|  | Disengagement and feedback | Disengaging the client from the program and seek feedback from the client for the purpose of evaluation |
| Evaluation and outcomes | Client satisfaction | Client satisfaction with case management program |
|  | Assessment of costs | Assessment of costs against the benefits achieved |
|  | Assessment of services provided | The quality and effectiveness of the services provided, participation in research activities, to improve practice, formal evaluation |
|  | Effectiveness in supporting the client | Case manager quality and effectiveness in supporting the client, level of case management to support client, improved quality of life, goals met etc. |
|  | Training | Continued professional training and development, supervision or mentoring from experienced case manager |
