# Supplementary material for: Implementation Outcomes and Their Determinants for Hospital‐Led Care Coordination Interventions Targeting Patients With Complex Care Needs: A Qualitative Systematic Review
Source: J Clin Nurs. 2025 Sep 15;35(3):1079–103. doi: 10.1111/jocn.70102 (PMC12862520; doi:10.1111/jocn.70102)
Supplement: Supplementary file 4 — Appendix S4: jocn70102‐sup‐0003‐AppendixS4.docx. [file JOCN-35-1079-s002.docx]

| **Category of study design** | **Methodological quality criteria** | Blignault et al. (2021) | Galarraga et al. (2021) | Gesell et al. (2019) | Green et al. (2023) | Horny et al. (2017) | Hudon et al. (2022) | Kahan et al. (2016) | Kimbell et al. (2018) | Lutz et al. (2020) | Markle-Reid et al. (2020) | McCreight et al. (2022) | Nurjono et al. (2019) | Orme et al. (2022) | Rocque et al. (2016) | Soto-Perez-de-Celis et al. (2021) | Wilcox et al. (2018) |
| --- | --- | --- | --- | --- | --- | --- | --- | --- | --- | --- | --- | --- | --- | --- | --- | --- | --- |
| **Screening questions (for all types)** | S1. Are there clear research questions? | Yes | Yes | Yes | Yes | Yes | Yes | Yes | Yes | Yes | Yes | Yes | Yes | Yes | Yes | Yes | Yes |
|  | S2. Do the collected data allow to address the research questions? | Yes | Yes | Yes | Yes | Yes | Yes | Yes | Yes | Yes | Yes | Yes | Yes | Yes | Yes | Yes | Yes |
|  | *Further appraisal may not be feasible or appropriate when the answer is 'No' or 'Can’t tell' to one or both screening questions.* |  |  |  |  |  |  |  |  |  |  |  |  |  |  |  |  |
| **1. Qualitative** | 1.1. Is the qualitative approach appropriate to answer the research question? | Yes | No |  | Yes |  | Yes | Yes | Can't tell | Yes | Yes | Yes | Yes |  |  |  |  |
|  | 1.2. Are the qualitative data collections methods adequate to address the research question? | Yes | Yes |  | Yes |  | Yes | Yes | Yes | Yes | Yes | Yes | Yes |  |  |  |  |
|  | 1.3. Are the findings adequately derived from the data? | Yes | Yes |  | Yes |  | Yes | Yes | Yes | Yes | Yes | Yes | Yes |  |  |  |  |
|  | 1.4. Is the interpretation of results sufficiently substantiated by data? | Yes | Yes |  | Yes |  | Yes | Yes | Yes | Yes | Can't tell | Yes | Yes |  |  |  |  |
|  | 1.5. Is there coherence between qualitative data sources, collection, analysis and interpretation? | Yes | Yes |  | Yes |  | Yes | Yes | Yes | Yes | Can't tell | Yes | Yes |  |  |  |  |
| **2. Quantitative randomized controlled trials** | 2.1. Is randomization appropriately performed? |  |  | Yes |  |  |  |  |  |  |  |  |  | Yes |  | Can't tell |  |
|  | 2.2. Are the groups comparable at baseline? |  |  | NA |  |  |  |  |  |  |  |  |  | Can't tell |  | Yes |  |
|  | 2.3. Are there complete outcome data? |  |  | NA |  |  |  |  |  |  |  |  |  | Yes |  | Yes |  |
|  | 2.4. Are the outcome assessors blinded to the intervention provided? |  |  | Yes |  |  |  |  |  |  |  |  |  | No |  | No |  |
|  | 2.5. Did the participants adhere to the assigned intervention? |  |  | Can't tell |  |  |  |  |  |  |  |  |  | Can't tell |  | Can't tell |  |
| **3. Quantitative non-randomised** | 3.1. Are the participants representative of the target population? |  |  |  |  | Yes |  |  |  |  | Yes |  |  |  |  |  |  |
|  | 3.2. Are measurements appropriate regarding both the outcome and intervention (or exposure)? |  |  |  |  | Yes |  |  |  |  | Yes |  |  |  |  |  |  |
|  | 3.3. Are there complete outcome data? |  |  |  |  | Yes |  |  |  |  | Yes |  |  |  |  |  |  |
|  | 3.4. Are the confounders accounted for in the design and analysis? |  |  |  |  | Yes |  |  |  |  | NA |  |  |  |  |  |  |
|  | 3.5. During the study period, is the intervention administered (or exposure occurred) as intended? |  |  |  |  | Can't tell |  |  |  |  | Yes |  |  |  |  |  |  |
| **4. Quantitative descriptive** | 4.1. Is the sampling strategy relevant to address the research question? |  |  |  | Yes |  | Yes |  | Yes |  |  | Yes | Yes |  | Can't tell |  | Can't tell |
|  | 4.2. Is the sample representative of the target population? |  |  |  | Yes |  | Yes |  | Yes |  |  | NA | Can't tell |  | Can't tell |  | Can't tell |
|  | 4.3. Are the measurements appropriate? |  |  |  | Yes |  | Yes |  | Yes |  |  | Yes | Yes |  | Yes |  | Yes |
|  | 4.4. Is the risk of nonresponse bias low? |  |  |  | Can't tell |  | Yes |  | Can't tell |  |  | NA | Can't tell |  | Yes |  | No |
|  | 4.5. Is the statistical analysis appropriate to answer the research question? |  |  |  | NA |  | Yes |  | Yes |  |  | NA | Yes |  | No |  | Yes |
| **5. Mixed methods** | 5.1. Is there adequate rationale for using a mixed methods design to address the research question? |  |  |  | No |  | Yes |  | No |  | Yes | Yes | No |  |  |  |  |
|  | 5.2. Are the different components of the study effectively integrated to answer the research question? |  |  |  | No |  | Yes |  | Can't tell |  | No | Yes | Yes |  |  |  |  |
|  | 5.3. Are the outputs of the integration of qualitative and quantitative components adequately addressed? |  |  |  | No |  | Yes |  | No |  | No | Yes | Yes |  |  |  |  |
|  | 5.4. Are divergences and inconsistencies between quantitative and qualitative results adequately addressed? |  |  |  | No |  | Yes |  | No |  | No | Yes | Yes |  |  |  |  |
|  | 5.5. Do the different components of the study adhere to the quality criteria of each tradition of the methods involved? |  |  |  | Yes |  | Yes |  | Yes |  | Yes | Yes | Yes |  |  |  |  |

NA Not Applicable
